# Rapid characterisation of the inherent dispersibility of respirable powders using dry dispersion laser diffraction

**DOI:** 10.1016/j.ijpharm.2013.02.034

**Published:** 2013-04-15

**Authors:** Sara Jaffari, Ben Forbes, Elizabeth Collins, David J. Barlow, Gary P. Martin, Darragh Murnane

**Affiliations:** a King's College London, Institute of Pharmaceutical Science, Stamford Street, London SE1 9NH, UK; b Pfizer Global R&D, Ramsgate Road, Sandwich, Kent CT13 9NJ, UK; c University of Hertfordshire, Department of Pharmacy, College Lane, Hatfield AL10 9AB, UK

**Keywords:** Dry powder inhaler (DPI), Laser diffraction analysis, Sympatec HELOS and RODOS, Cohesion, Inhalation, De-agglomeration

## Abstract

Understanding and controlling powder de-agglomeration is of great importance in the development of dry powder inhaler (DPI) products. Dry dispersion laser diffraction measures particle size readily under controlled dispersing conditions, but has not been exploited fully to characterise inherent powder dispersibility. The aim of the study was to utilise particle size-dispersing pressure titration curves to characterise powder cohesivity and ease of de-agglomeration. Seven inhaled drug/excipient powders (beclometasone dipropionate, budesonide, fluticasone propionate, lactohale 300, salbutamol base, salmeterol xinafoate and tofimilast) were subjected to a range of dispersing pressures (0.2–4.5 Bar) in the Sympatec HELOS/RODOS laser diffractometer and particle size measurements were recorded. Particle size-primary pressure data were used to determine the pressures required for complete de-agglomeration. The latter were employed as an index of the cohesive strength of the powder (critical primary pressure; CPP), and the curves were modelled empirically to derive the pressure required for 50% de-agglomeration (DA_50_). The powders presented a range of CPP (1.0–3.5 Bar) and DA_50_ (0.23–1.45 Bar) which appeared to be characteristic for different mechanisms of powder de-agglomeration. This approach has utility as a rapid pre-formulation tool to measure inherent powder dispersibility, in order to direct the development strategy of DPI products.

## Graphical abstract

**Figure:**
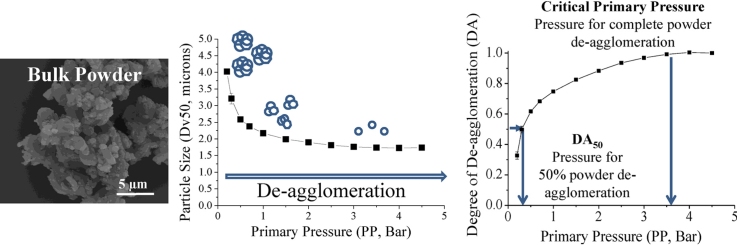

## 1 Introduction

Drug deposition within the respiratory tract is dependent on the delivery of particles with an aerodynamic size of <5 μm (Shekunov et al., 2003; Usmani et al., 2005). The intrinsic cohesivity of such fine and often irregularly shaped particles means that there is a tendency for particles to agglomerate (Adi et al., 2011). Micronised drug particles are therefore often formulated with large carrier particles as a means of aiding powder dispersion (Telko and Hickey, 2005). During delivery it is essential that attractive drug–drug (cohesive) and drug–carrier (adhesive) forces are overcome and particles are restored to their primary de-agglomerated state (Telko and Hickey, 2005). Although device and patient factors affect de-agglomeration, the inherent dispersibility of the powder is a major factor dictating the ease of agglomerate dispersal and hence the delivered dose. Any imbalance of the cohesive and adhesive forces may adversely affect de-agglomeration, resulting in poor aerosolisation of the powder formulation (Begat et al., 2004b).

The ease of de-agglomeration of powders may be predicted using indirect methods that measure inter-particulate forces. These include highly technical single-particle techniques such as atomic force microscopy (AFM) (Jones et al., 2008; Begat et al., 2004a) and bulk techniques such as inverse gas chromatography (IGC) (Das et al., 2009c; Jones et al., 2012; Tong et al., 2006). AFM only measures a small proportion of the powder and provides potentially poor representation of bulk properties (Bunker et al., 2005), while IGC requires large quantities of material and long analysis times. Specially designed de-agglomeration rigs allow agglomerate break-up to be studied under controlled levels of turbulence or impaction, but need to be used in tandem with a particle size spectrometer or cascade impactor (Kurkela et al., 2008; Voss and Finlay, 2002). Impactor methods measure aerosol dispersion directly; the emitted dose provides an index of powder entrainment, and the fine particle mass is indicative of de-agglomeration efficiency (Louey et al., 2006). Although impactor methods are excellent for the quality control testing of inhalation products, the analytical procedures involved are labour-intensive and time consuming, making the methods unsuitable for rapid screening of powder formulation dispersibility during the early stages of product development (Marriott et al., 2006).

Dry dispersion laser diffraction (LD) is increasingly used to study powder de-agglomeration. When powder is delivered to the sizer from a static bed, the powder is subjected to minimal disturbance to the powder structure and an assessment of inherent dispersibility can be made. Pressure titration, whereby size measurements are made under different levels of dispersing shear, is a requirement of method development for LD particle sizing in the dry state to define those instrument parameters that provide accuracy and precision of sizing measurements (ISO 13320: 1990). Under well-controlled dispersing conditions, changes in the measured particle size distribution (PSD) of an aerosolised plume provide an indication of agglomerate strength and degree of de-agglomeration (Adi et al., 2006; Ghoroi et al., 2012). Despite never having been substantiated in a systematic study of pharmaceutical powders, the gradient of measured particle size over a dispersion pressure/airflow range has been suggested as an indicator of powder dispersibility (Adi et al., 2006, 2008; Chiou et al., 2008; Ghoroi et al., 2012; Kaye et al., 2009). Titration experiments have been conducted for this purpose, but studies have not been validated using a wide range of sample powders nor have they involved the generation of standardised parameters for describing de-agglomeration. Where titration data have been related more fully to aerosolisation parameters (e.g. Behara et al., 2011a,b; Chiou et al., 2008) the studies have determined the particle size of the powders following actuation from either inhaler devices or dry powder dispersers, which contribute themselves to the de-agglomeration of the powder under investigation. During the early stages of inhaled product development, information is required about the fundamental dispersion behaviour of the drug powder in order to guide formulation and device designs. If dry dispersion LD were qualified as a technique for measuring particle size-dispersing pressure curves in a systematic manner, for powders delivered from a static bed, this would allow the inherent dispersibility of dry powders to be assessed reliably in the absence of device/disperser effects.

The aim of this work was to develop a rapid method to quantify the de-agglomeration of dry powders from a static bed as a measure of inherent powder dispersibility. An optimised methodology based on LD was developed, standardised and used to measure particle size-dispersing pressure titration curves for a number of inhaled drug/excipient powders. These data were used to derive parameters indicative of powder cohesivity and ease of de-agglomeration.

## 2 Materials and methods

### 2.1 Materials

The inhalation powders used in the study were beclometasone dipropionate (BDP; Pharm Dev Europe, GWRD, BN. WC60329), budesonide (Bud; LGM Pharma, USA, BN. U0015/1V040), fluticasone propionate (FP; LGM Pharma, USA, BN. 458763), Lactohale 300 (LH300; Frieslands Foods, Domo, The Netherlands, BN. 6125224/S), salbutamol base (SB; Pharm Dev Europe, GWRD, BN. WC46269), salmeterol xinafoate (SX; Vamsi Labs, India, BN. SX-0081010), and tofimilast (Tof; Pfizer Ltd, PGRD Sandwich Laboratories, UK). Cyclohexane was purchased from VWR International (France), methanol and sorbitan monooleate 80 (Span 80) were from Sigma Aldrich Ltd (UK), hexane was from Fisher Scientific (Loughborough, UK), and Span 85 and Tween 80 were from Merck (Schuchardt, Germany).

### 2.2 Methods

#### 2.2.1 Particle size measurements by liquid dispersion laser diffraction

Laser diffraction particle sizing was carried out using a Malvern Mastersizer X (Malvern Instruments Ltd, UK) fitted with a 100 mm focal length lens (0.5–180 μm) and an MS7 magnetically stirred cell. Saturated solvent dispersants were prepared by sonicating for 30 min followed by overnight stirring. Approximately 1 mg of powder was added to 2 mL filtered dispersant (0.2 μm cellulose acetate syringe filter, Gema Medical S.L., Spain) and sonicated (Sonicleaner, DAWE, Ultrasonics Ltd, USA). A background reading was taken and the suspension was added to the sample cell until the obscuration was ∼10–30%. Following equilibration (30–60 s), ten individual measurements were taken for *n* = 3 samples to obtain particle size measurements (*D*_v10_, *D*_v50_, and *D*_v90_, corresponding to the particle size below which 10%, 50% and 90% of the particles by volume are smaller than, and the volume mean diameter, VMD, the volume weighted mean particle size of the sample) calculated using Fraunhofer theory. A summary of the dispersant, sonication time, stir setting, sweeps, presentation and equilibration time used to size each powder are provided in Table 1.

#### 2.2.2 Scanning electron microscopy (SEM)

Powder samples were transferred onto glass cover-slips placed on adhesive carbon tabs (G3347N, Agar Scientific, Essex, England) which were mounted onto aluminium pin stubs (0.5 in.; G301, Agar Scientific Ltd, Essex, England). Samples were sputter coated with gold for 2 min to achieve a thickness of approx. 15–20 nm using a K550X sputter coater (Emitech, Quorum Technologies Limited, West Sussex, England). Particle morphology was viewed using a Quanta 200F field emission scanning electron microscope (FEI UK Ltd, Cambridge, England) operated at 10 kV in low vacuum mode and a working distance of 10 mm.

#### 2.2.3 Particle size measurements by dry dispersion laser diffraction

Particle size measurements were made using a Sympatec HELOS/RODOS (Sympatec GmbH, Clausthal-Zellerfel, Germany) employing the rotary feeder and R3 lens (0.9–175 μm). Powder was hand-filled into the u-shaped groove of the rotating table to cover a length of approximately 1 cm. The sample passed under a plough scraper and roller to remove any excess and was subsequently drawn up into the dispersing line via the protruding aspiration tube from a static bed. During sample delivery the rotating table was maintained at a constant rotation setting of 20%. The measurement was set to trigger when the optical concentration (*C*_opt_) exceeded 1.1% and cease when the *C*_opt_ fell below 1% for 5 s (or 60 s real time). The timebase was 100 ms and a forced stability of ‘4’ was applied. The primary pressure (PP) was manually set using the adjustment valve in the range 0.2–4.5 Bar and three measurements were taken at each pressure setting using freshly loaded powder. PSDs (*D*_v10_, *D*_v50_, *D*_v90_ and VMD) were calculated using Fraunhofer theory and analysed in WINDOX 4.0 software. Particle size measurements for a complete titration curve were made on a single day.

#### 2.2.4 Critical primary pressure

The pressure at which the particle size-primary pressure profile reached a plateau was considered to represent the pressure required to overcome the interactive forces holding agglomerates together and therefore provide a measure of the cohesivity of the powder. The critical primary pressure (CPP) was derived by calculating a difference ratio (*d*_*r*_) using Eq. (1), where the *D*_v50_ is the geometric median diameter at a given primary pressure (mean of *n* = 3 measurements), and PP1 and PP2 are two consecutive primary pressures (PP2 > PP1). The CPP was assigned when *d*_*r*_ was in the range −0.06 < *d*_*r*_ > 0.06 for three consecutive measurements (the accepted coefficient of variance for *D*_v50_ values in particles of this size range when validating a particle size methodology according to ISO 13320: 1990):

(1) $$d_{r}=\frac{D_{v50}{\text{PP}}_{1}-D_{v50}{\text{PP}}_{2}}{D_{v50}{\text{PP}}_{1}}$$

#### 2.2.5 Ease of de-agglomeration

A method of data analysis was developed to derive a parameter for ease of de-agglomeration. The particle size data were normalised to account for differences in the primary particle size of each powder. The particle size at each primary pressure (*D*_*x*_) was expressed as a proportion of the fully dispersed particle size (*D*_H_, the *D*_v50_ measured at the highest PP) according to Eq. (2). The degree of de-agglomeration (DA) was therefore a measure of the extent of de-agglomeration achieved at any particular pressure, with complete dispersion of the powder to primary particles at DA = 1.

(2) $$\text{DA}=\frac{D_{H}}{D_{x}}$$

**Fig. 1:**
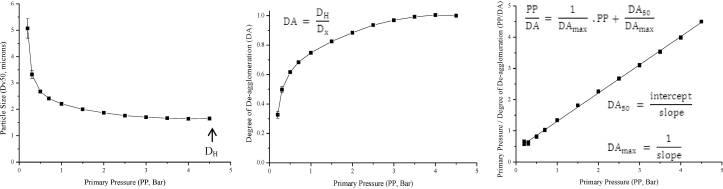
A schematic of the de-agglomeration analysis employed using beclometasone dipropionate (BDP) data, where *D*_*H*_ is the *D*_v50_ at the highest primary pressure (PP), *D*_*x*_ is the measured *D*_v50_ at a particular PP, DA is the degree of de-agglomeration, DA_50_ is the PP for 50% de-agglomeration and DA_max_ is the maximum DA.

The de-agglomeration data exhibited rectangular hyperbolas and were empirically modelled. When fitted to a linearised Freundlich equation the linearity was poor (*R*^2^ = 0.69–0.92), but the modified Michaelis–Menten equation linearised using the Hanes–Woolf approach (Fig. 1, Eq. (3)) provided an excellent fit. The maximum degree of de-agglomeration (DA_max_) was obtained from the asymptote degree of de-agglomeration, calculated using the slope of the fitted regression line (Fig. 1). The DA_50_ was derived from the intercept and slope of the regression line to obtain the primary pressure required to achieve 50% de-agglomeration (Fig. 1). The DA_50_ was chosen as it could be derived from the regression line of the linearised data without further data manipulation or errors of interpolation, such as would result from interpolation.

(3) $$\frac{\text{PP}}{\text{DA}}=\frac{1}{{\text{DA}}_{\text{max}}},\;\text{PP}+\frac{{\text{DA}}_{50}}{{\text{DA}}_{\text{max}}}$$

## 3 Results

### 3.1 Particle size of the powders by liquid dispersion

The liquid dispersion particle size was measured to determine the fully dispersed particle size in a liquid medium independent of the dry dispersion laser diffraction technique. To maintain consistency between sizers, Fraunhofer theory was used in all particle size calculations. The results confirmed that all the powders possessed a PSD within the micron size range (Table 2), with *D*_v50_ values ranging from 1.44 ± 0.16 μm for SB to 3.74 ± 0.41 μm for LH300. In all instances the *D*_v50_ was less than 4 μm and therefore of a size typical of that used for inhaled delivery.

### 3.2 Particle morphology

**Fig. 2:**
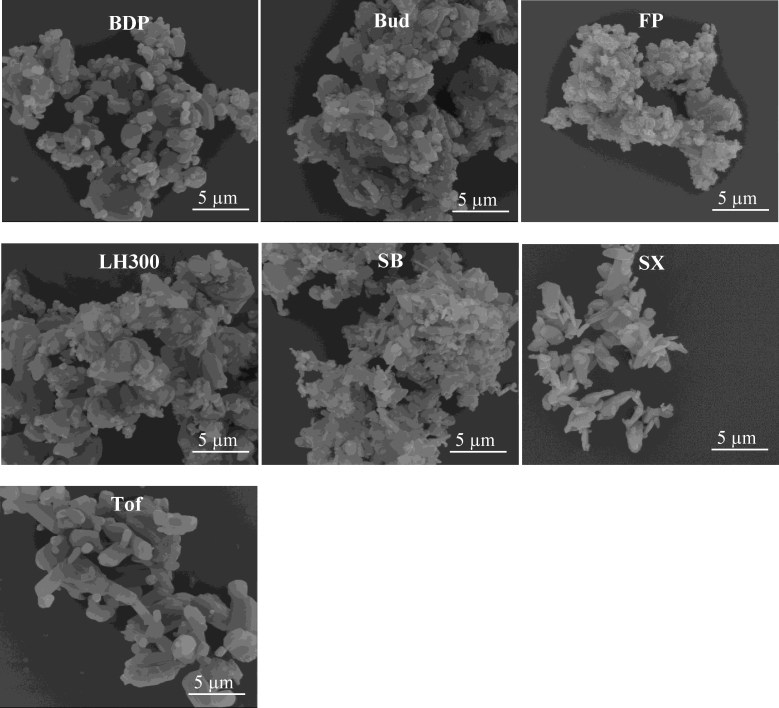
Scanning electron micrograph images of beclometasone dipropionate (BDP), budesonide (Bud), fluticasone propionate (FP), lactohale 300 (LH300), salbutamol base (SB), salmeterol xinafoate (SX) and tofimilast (Tof) at 10,500× magnification.

SEM imaging further confirmed that each powder was composed of micron-sized particles (Fig. 2). All the powders were agglomerated and exhibited a range of agglomerate sizes. The agglomerates were also observed to be in association with each other, suggesting that inter-agglomerate interactions occurred leading to the formation of larger agglomerated structures within the powder.

### 3.3 Effect of primary pressure on the particle size distribution following dry dispersion

**Fig. 3:**
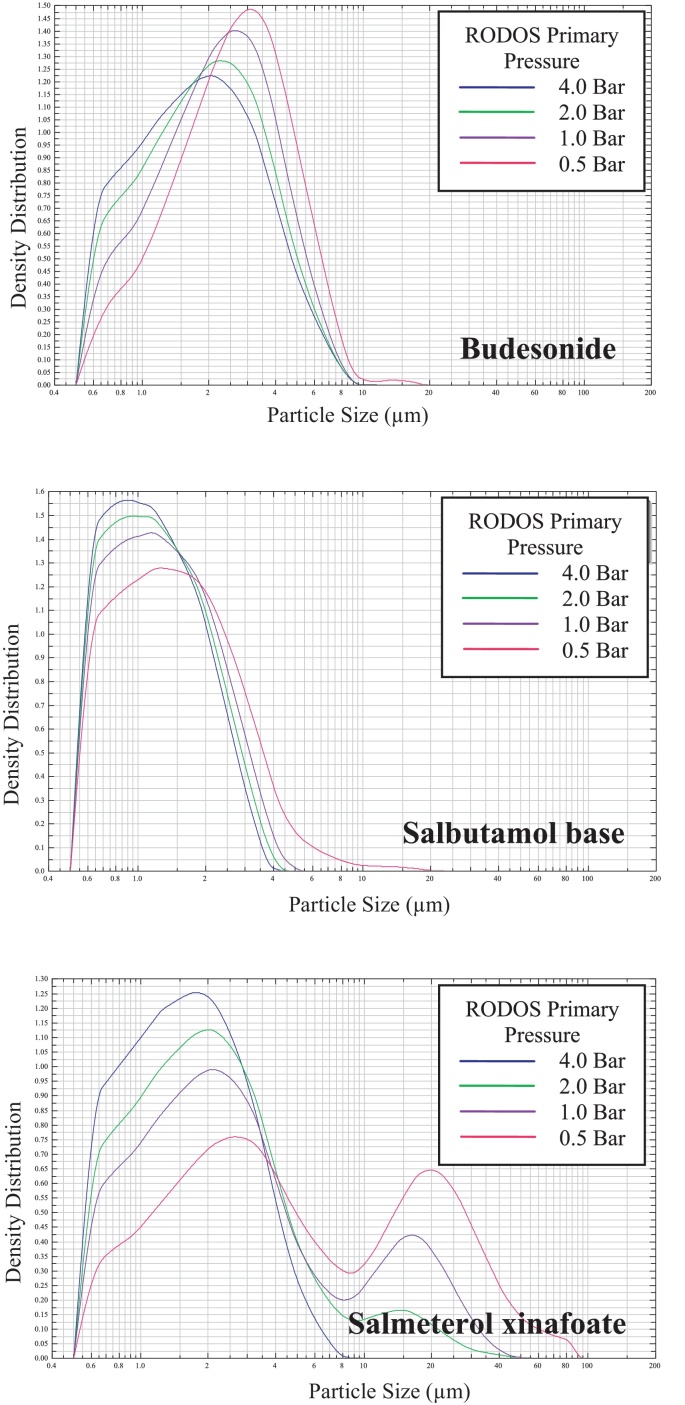
The particle size distribution of budesonide (Bud), salbutamol base (SB), and salmeterol xinafoate (SX) at 0.5, 1.0, 2.0 and 4.0 Bar primary pressure measured by Sympatec HELOS/RODOS dry dispersion laser diffraction (*n* = 1 measurement shown).

A shift towards smaller particle sizes was seen in the PSD for all the powders as primary pressure increased (Fig. 3). For some powders i.e. BDP, Bud (Fig. 3), LH300 and Tof, a small shoulder in the curve was seen due to very fine particles. This shoulder could arise as a result of particle fracture at high dispersing pressures. SB showed no shift and only a narrowing in the PSD (Fig. 3) indicating a narrow distribution in particle/agglomerate sizes even under a low level of dispersing pressure (i.e. a powder which readily disperses). FP and SX (Fig. 3) demonstrated bimodal distributions at low primary pressures, which were attributed to the powder containing a population of readily dispersible fine particles/agglomerates and a second population of larger cohesive agglomerates. At high primary pressures a shift of the fine particle peak occurred corresponding to an increase in the fine particle fraction (FPF) of smaller particles and a tendency to eliminate large agglomerates.

**Fig. 4:**
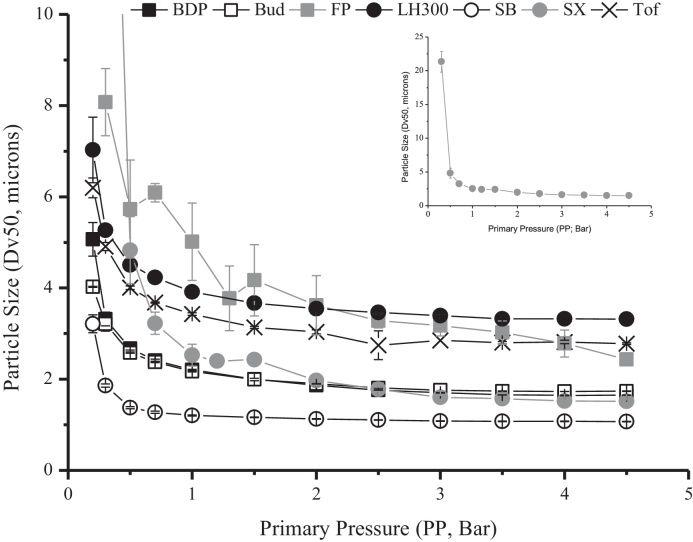
The particle size-primary pressure (PP) profiles of the inhaled powders measured by Sympatec HELOS/RODOS dry dispersion laser diffraction (mean ± SD, *n* = 3). Note: the profiles of beclometasone dipropionate (BDP) and budesonide (Bud) overlap; the inset graph is the particle size-primary pressure profile of salmeterol xinafoate (SX) which had the largest measured size at the lowest PP (data point omitted from main graph).

When the *D*_v50_ was plotted as a particle size-primary pressure profile (Fig. 4) all the powders exhibited a reduction in particle size until a plateau size was reached. The plateau particle size was comparable to that measured using liquid dispersion, indicating that complete dispersal was achieved. The pre-plateau profiles of the particle size-primary pressure curves differed, indicating powder-specific de-agglomeration behaviour (Fig. 4). Traditional pressure titration analysis during validation of particle sizing methods fails to investigate this pre-plateau behaviour despite the wealth of information, pertinent to inhaled product development, which it can offer regarding the de-agglomeration process. The high degree of variability of SX and FP particle size at low dispersing pressures (i.e. large standard deviations) indicated heterogeneity in agglomerate size and strength in these powders. SB had the smallest and SX the largest agglomerate size at low dispersing pressure. Some of the powders reached their plateau particle size at low primary pressures, e.g. SB, whereas for other powders complete de-agglomeration required higher dispersing pressures e.g. FP and Tof.

### 3.4 Cohesivity and ease of de-agglomeration

The CPP indicated that the powders varied in their cohesive strength from 1.0 Bar for SB to 3.5 Bar for SX indicating the least and most cohesive powders, respectively (Table 3). It was therefore possible to rank the powders from low to high cohesivity as follows: SB < Bud = LH300 < BDP < FP = Tof < SX.

**Fig. 5:**
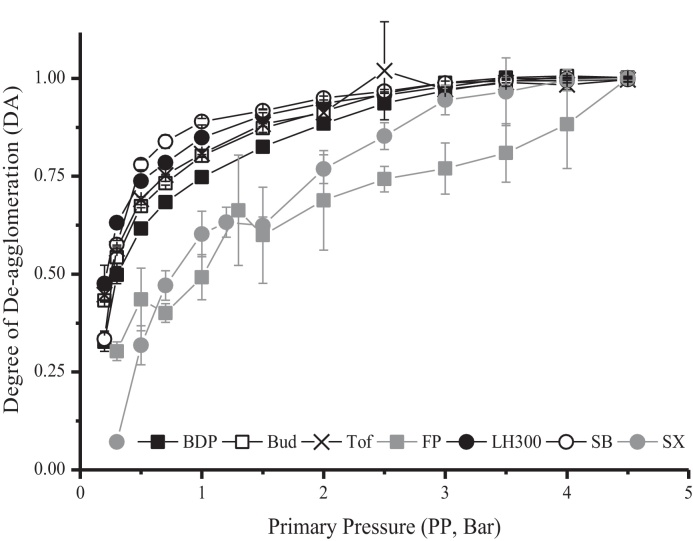
The de-agglomeration profiles of the inhaled powders beclometasone dipropionate (BDP), budesonide (Bud), fluticasone propionate (FP), lactohale 300 (LH300), salbutamol base (SB), salmeterol xinafoate (SX) and tofimilast (Tof) measured by Sympatec HELOS/RODOS dry dispersion laser diffraction (mean ± SD, *n* = 3).

Distinct de-agglomeration profiles were obtained for the powders following normalisation (Fig. 5). Linear transformations provided excellent fits for all the powders (*R*^2^ > 0.99 except for SX *R*^2^ = 0.97 and FP *R*^2^ = 0.91, Table 3). Calculated DA_max_ values reached 1.0, as would be expected for complete dispersion of the powders based on the normalisation data analysis approach adopted (Table 3). The DA_max_ also provided a further measure of the degree of fit of each data set to the model. The theoretical DA_max_ was 1.0, and powders showing the greatest deviation in this parameter (i.e. SX and FP) also had the poorest linearity owing to them possessing the largest variability (i.e. highest standard deviations) for replicate particle size measurements at low dispersing pressures (Fig. 4). The DA_50_, a measure of the relative ease of powder de-agglomeration (Table 3, Fig. 5), indicated that SX and FP de-agglomerated the least readily (DA_50_ = 1.45 and 1.15 Bar, respectively) and Bud, LH300, SB and Tof dispersed very readily (DA_50_ = 0.32, 0.23, 0.25, 0.28 Bar, respectively). BDP was an intermediate disperser (DA_50_ = 0.44 Bar).

## 4 Discussion

The ultimate goal of dry powder inhaler product development is to maximise the fraction of powder emitted upon inhalation by the patient which is of a respirable size (<5 μm). This requires the powder to be readily fluidisable and dispersible from a static powder bed. The formulation development process requires a knowledge of the de-agglomeration behaviour of the bulk micronised material, in order to select the required formulation components and manufacturing approach, to overcome the inherently cohesive nature of the powders. The aim of this work was to develop a rapid and systematic laser diffraction-data analytical method to characterise the de-agglomeration behaviour of potentially inhalable powders.

The liquid dispersed particle size (Table 2) and SEM images (Fig. 2) confirmed that the powders were micronised and of a size suitable for inhaled delivery, each having a *D*_v50_ less than 4 μm. These particle sizes corresponded with those obtained using dry dispersion under high primary pressure, verifying that complete de-agglomeration of the particles occurred under these conditions. Although there were significant differences (*p* < 0.05, unpaired students *t*-test) between the sizes measured using dry and liquid dispersion (except for LH300 and Tof), the absolute magnitude of the differences was small. For example, SX was found to have a *D*_v50_ of 2.05 ± 0.12 μm and 1.51 ± 0.03 μm in the liquid and dry dispersed state, respectively (Table 2). The dry dispersion size was consistently smaller than the liquid dispersed size; this may arise from high dispersing pressures in the Sympatec causing a degree of particle attrition/erosion due to high gas velocities and impactions in the dispersing line (Leschonski et al., 1984; Ghoroi et al., 2012). Differences in particle size can also arise as a consequence of the use of different laser diffractometers and techniques. These include the specific numerical algorithm used to analyse diffraction data (Etzler and Deanne, 1997), the potential for dispersion in a liquid to alter the particle shape, volume, and dissolution characteristics of the sample, and also the orientation of particles in the sample cell (Berthold et al., 2000).

DPI devices generally require powder de-agglomeration to occur under relatively low dispersion forces (Shekunov et al., 2003). When using low primary pressures for dry dispersion sizing, agglomerates are often present and are measured in tandem with primary particles. The *D*_v50_ of the powders ranged from 21.35 ± 1.52 μm for SX to 1.86 ± 0.03 μm for SB at 0.3 Bar dispersing pressure. The powders therefore had different tendencies for agglomeration and different agglomerate strengths under equivalent low level shear conditions. For example, SB consisted predominantly of individual particles and small SB–SB agglomerates whereas SX consisted of large SX–SX agglomerates. Large SX agglomerates have been reported at low dispersing pressures, and are associated with low FPFs and high throat and pre-separator deposition following aerosolisation into cascade impactors (Adi et al., 2008; Shekunov et al., 2003). Highly cohesive powders tend to entrain as large agglomerates (Shur et al., 2008; Tuley et al., 2008), and the high tensile strength (which may be associated with large, densely packed agglomerates) requires high levels of dispersing shear to overcome inter-particulate interactions (Adi et al., 2006). Although the agglomeration state at low primary pressures cannot in isolation be used as an indicator of fundamental dispersibility, it allows for a qualitative distinction between powders of different cohesivity and structure following the application of low levels of shear.

The shape of the particle size-primary pressure profile provided a mechanistic insight into the de-agglomeration process. For example, the rapid drop in the *D*_v50_ of SB under the application of a dispersing pressure, and rapid attainment of the plateau particle size, indicated efficient de-agglomeration to near complete agglomerate dispersal occurring very readily under the application of shear. However, for FP, the drop in the *D*_v50_ was more progressive suggesting a more gradual de-agglomeration process. Previously, flat gradients in particle size-dispersing shear profiles have been attributed to powders showing little agglomeration and more efficient dispersion (Adi et al., 2006; Begat et al., 2004b; Ghoroi et al., 2012; Kaye et al., 2009). By using multiple dispersing pressures over a wide pressure range, and testing a number of powders, it is apparent that the entire curve and not solely the gradient must be considered. For example, when the complete curve was empirically modelled, FP was shown to disperse less readily than SB. Furthermore, SX and FP were found to both have poor dispersibility despite having different particle size-dispersing pressure profiles. The profile can also provide insight into powder structure; for example, high standard deviations were obtained in the measured agglomerate size at each dispersing pressure for FP. This highlighted agglomerate size heterogeneity during dispersion, such that under a given level of shear there will be mixed populations of agglomerate and de-agglomerated particle sizes. This may have implications for the achievable fine particle dose, and its reproducibility following delivery, and therefore knowledge of this will allow for rational formulation/delivery approaches.

Inter-particulate cohesive forces are difficult to characterise due to heterogeneity in particle properties such as size, shape, surface area and morphology (Telko and Hickey, 2005). They are also influenced by powder packing/tensile strength (Shur et al., 2008), external factors such as forces experienced during handling (Podczeck et al., 1994) and relative humidity (Das et al., 2009a,b). The CPP, derived from the particle size-dispersing pressure curve, reflects the dispersing pressure required to overcome the cohesive interactive forces between particles, and is determined by the most cohesive agglomerates which resist break-up at low pressures. For full de-agglomeration to occur, sufficient energy must be provided to overcome these most cohesive forces. The CPP values revealed differences between the powders in the magnitude of inter-particulate forces of the most cohesive particles/agglomerates (Table 3) allowing the powders to be ranked, and further identifying that different formulation/device approaches would be necessary to achieve complete de-agglomeration of these powders. A qualitative interpretation of the particle size-dispersing stress titration curves presented by Shekunov et al. (2003) suggests that SX generated from precipitation in supercritical fluids (S-SX) would have a lower CPP than micronised SX (M-SX). S-SX particles also had lower surface energy and generated higher FPFs than M-SX (Shekunov et al., 2003). The CPP would therefore provide a further parameter, obtainable in a rapid and straightforward manner, indicating that lower dispersing stresses are required for these particles to achieve de-agglomeration.

The DA (degree of de-agglomeration) was calculated at every dispersing pressure in order to construct de-agglomeration profiles. At low dispersing pressures e.g. 0.3 Bar, the DA of the powders was low (e.g. DA = 0.30 ± 0.02 for FP and DA = 0.07 ± 0.01 for SX). A dispersing pressure of 0.1 Bar is approximately equivalent to the viscous shear stress (*τ*_S_) across some DPI devices such as the Clickhaler (Shekunov et al., 2003). The DA would therefore predict that a significant proportion of the powder (e.g. >30% for FP) would remain agglomerated and therefore be unavailable for deposition in the target regions of the lungs following delivery. When sized following actuation from an inhaler device, significant linear correlations have been observed between laser diffraction and cascade impactor FPFs (Marriott et al., 2006; Martin et al., 2006; Zeng et al., 2006). The DA can therefore serve as a useful aid in the development of dry powder inhaler products by providing a rapid indication of the formulation and device characteristics that may be required, based on the inherent dispersibility of the powder, in order to overcome cohesive forces and achieve the optimal FPF.

The parameter DA_50_, the dispersing pressure for 50% de-agglomeration, provides a measure of how readily a powder disperses. The value was derived from the intercept and slope of the regression line of the linearised de-agglomeration profiles, and therefore takes into account the shape of the individual de-agglomeration curve. Being directly determined from linear regression parameters (gradient and intercept), the use of the DA_50_ avoids errors of interpolation which would be achieved with other indices (e.g. 10th percentile). The powders could be characterised into those that were poor (SX and FP, DA_50_ = 1.45 and 1.15 Bar, respectively), intermediate (BDP DA_50_ = 0.44 Bar) and good (Bud, LH300, SB, Tof, DA_50_ = 0.32, 0.23, 0.25, 0.28 Bar, respectively) dispersers.

Together, the shape of the particle size-primary pressure profile, the CPP and DA_50_ values allow the de-agglomeration mechanisms of the powders to be postulated. The absolute difference between the DA_50_ and the CPP can be used to estimate the degree of heterogeneity in cohesive forces of the sample powder. A low CPP and DA_50_ were obtained for LH300, SB and Bud, and resulted in a powder that dispersed very readily, and de-agglomerated completely, under a range of shear stresses. The PSDs of these powders were mono-model, consistent with low cohesive forces between the particles and fluidisation via an erosion mechanism, where a stream of de-agglomerated particles are continually entrained into the airflow (Shur et al., 2008; Tuley et al., 2008). In contrast, when CPP and DA_50_ were both large, e.g. SX and FP, an explosive de-agglomeration is indicated. At low shear the powders undergo very little dispersion, due to the entrainment of large agglomerates (Shur et al., 2008; Tuley et al., 2008), some of which are so tightly associated that a bi-modal PSD is observed indicative of a distinct population of tightly associated agglomerates. When higher levels of shear, equal to or greater than the CPP, are applied, instantaneous de-agglomeration to primary particles occurs as the inter-particulate forces are overcome.

One of the powders, Tof, had a high CPP but a low DA_50_. This may suggest heterogeneity in the magnitude of inter-particulate forces as some powder agglomerates will break-up readily (hence a low DA_50_) but others will require a much higher dispersing pressure (hence a high CPP) for de-agglomeration. Powders with inter-particulate force homogeneity would conversely have similar DA_50_ and CPP values, with complete dispersion of agglomerates occurring upon the application of the appropriate level of shear able to overcome intra-agglomerate interactive forces.

A key advantage of the laser diffraction technique as developed and described in this study is therefore its ability to account for powder heterogeneity, and in doing so provide a more accurate representation of powder properties compared to many existing techniques. Single particle techniques such as AFM can provide useful insights e.g., budesonide has been reported to display stronger cohesive interactive forces than lactose (Begat et al., 2004a), whereas those of salmeterol xinafoate and fluticasone propionate are indicated to be of comparable magnitude (Young et al., 2004). However, since AFM by its nature can only employ a small number of isolated particles, then extrapolation of the results to the aerosolisation behaviour of a powder bed, where the bulk cohesive properties of the powder are clearly important, should only be effected with caution. For example, if only the most cohesive particles are sampled, where a distribution of particles is likely due to inter- and intra-batch variability in properties (Feeley et al., 1998; Price, 2011), there may be an overestimation of the cohesive force of a bulk powder.

Techniques such as IGC, when using traditional infinite dilution, probes only the most active surface sites and in a similar manner to AFM can be misleading with regards to overall powder cohesive strength; reported values in the literature can vary e.g. the dispersive surface energy (*γ*_D_) of SX is 41–48 mJ m^−2^ (Das et al., 2009c; Tong et al., 2002). Finite dilution is able to take into account surface heterogeneity but analysis is lengthy. Differences in the distribution of high energy sites have been identified (Tong et al., 2002; Das et al., 2012). Furthermore, heterogeneity in particle properties leading to non-homogenous powder strength distributions has been attributed to different de-agglomeration behaviours between lactose samples (Das et al., 2012).

Being a bulk measurement technique, dry dispersion LD, if conducted within a controlled environment, is therefore able to account for every factor which may influence de-agglomeration, including particulate, bulk, and external factors. Furthermore, there is little powder manipulation prior to analysis, which results in minimal disturbance to the powder structure/packing, and the analysis times are short. These advantages provide a convenient, accessible and highly relevant tool in powder characterisation and the necessary preceding step in formulation development.

## 5 Conclusion

The de-agglomeration behaviour of a number of chemically distinct micronised powders for inhalation was evaluated using a dry dispersion laser diffraction technique. The bulk powders were characterised with respect to their cohesive strength (CPP) and ease of de-agglomeration (DA_50_). The relative CPP and DA_50_ enabled powder structure and de-agglomeration mechanisms to be postulated. A strong feature of the approach is the extraction of meaningful parameters from bulk powders rather than the generation of descriptors relating to individual particles, thereby incorporating powder heterogeneity effects which may be lost through the use of single particle measures such as AFM. The method also allowed for rapid analysis times with little powder manipulation to disturb powder structure/packing, which compares favourably with, for example, IGC. The measurements also take into account all types of interactive force and their heterogeneity, using a single methodological approach. The ability to characterise the powders independently of any formulation or device distinguishes the method as a powder screening tool for inherent powder dispersibility that may be of use in a variety of pharmaceutical applications.

## Figures and Tables

**Table 1:** A summary of the liquid dispersion laser diffraction parameters used in particle sizing of the powders.

Powder	Dispersant (%, w/v)	Sonication time (min)	Stir setting	Sweeps	Equilibration time (s)
BDP^a^	1.0% Span 80 in cyclohexane	5	3	3500	60
Bud^b^	0.1% Span 80 in cyclohexane	15	3	3500	60
FP^c^	0.1% Span 80 in cyclohexane	2.5	3	3500	60
LH300^d^	0.1% Span 80 in cyclohexane	1	3	3500	30
SB^d^	1.0% Span 85 in hexane	1	3	3500	30
SX^c^	0.5% span 80 in cyclohexane^c^	5	3	2500	60
Tof	0.05% Tween 80 in 5% (v/v) methanol and water	5	5	2500	60

a Zeng et al. (2000).

b Buttini et al. (2008).

c Murnane (2009).

d Tang (2008).

**Table 2:** The geometric particle size (*D*_v10_, *D*_v50_, *D*_v90_, VMD) of the powders assessed by dry dispersion laser diffraction at 4.5 Bar primary pressure (*n* ≥ 3, mean ± SD) and liquid dispersion laser diffraction (*n* = 3, mean ± SD).

Powder	Dispersion	*D*_v10_ (μm)	*D*_v50_ (μm)	*D*_v90_ (μm)	VMD (μm)	Span
BDP	Dry	0.70 ± 0.00	1.65 ± 0.00	3.50 ± 0.00	1.90 ± 0.01	1.70
	Liquid	1.20 ± 0.02	2.62 ± 0.24	4.75 ± 0.71	2.87 ± 0.32	1.36

Bud	Dry	0.70 ± 0.00	1.74 ± 0.00	4.08 ± 0.01	2.12 ± 0.00	1.94
	Liquid	1.02 ± 0.02	2.17 ± 0.03	4.39 ± 0.03	2.49 ± 0.02	1.55

FP	Dry	0.88 ± 0.03	2.44 ± 0.11	5.75 ± 0.23	3.01 ± 0.13	1.90
	Liquid	1.25 ± 0.01	2.81 ± 0.03	5.18 ± 0.13	3.06 ± 0.05	1.40

LH300	Dry	0.91 ± 0.01	3.32 ± 0.01	8.26 ± 0.02	4.06 ± 0.01	2.22
	Liquid	1.78 ± 0.08	3.74 ± 0.41	6.57 ± 1.14	4.02 ± 0.52	1.28

SB	Dry	0.60 ± 0.01	1.07 ± 0.01	2.17 ± 0.01	1.25 ± 0.01	1.46
	Liquid	0.78 ± 0.08	1.44 ± 0.16	2.51 ± 0.29	1.57 ± 0.17	1.19

SX	Dry	0.67 ± 0.00	1.51 ± 0.03	3.47 ± 0.03	1.84 ± 0.01	1.83
	Liquid	0.81 ± 0.01	2.05 ± 0.12	4.33 ± 0.25	2.39 ± 0.18	1.71

Tof	Dry	1.13 ± 0.02	2.78 ± 0.02	5.48 ± 0.01	3.08 ± 0.01	1.56
	Liquid	0.87 ± 0.01	2.62 ± 0.02	4.98 ± 0.06	2.81 ± 0.02	1.57

**Table 3:** The linearity (*R*^2^), primary pressure for 50% de-agglomeration (DA_50_), maximum degree of de-agglomeration (DA_max_) and critical primary pressure (CPP) of the powders deduced from dry dispersion laser diffraction.

Powder	*R*^2^	DA_50_ (Bar)	DA_max_	CPP (Bar)
BDP	0.9990	0.44	1.11	2.5
Bud	0.9995	0.32	1.08	2.0
FP	0.9049	1.15	1.13	3.0^a^
LH300	0.9997	0.23	1.06	2.0
SB	0.9979	0.25	1.06	1.0
SX^b^	0.9711	1.45	1.35	3.5
Tof	0.9964	0.28	1.07	3.0

a Measured size unchanged for 2 consecutive dispersing pressures only, 3.0 and 3.5 Bar.

b Particle size data for the lowest PP (i.e. 0.3 Bar) omitted due to poor linearity with these data included (*R*^2^ = 0.4532).
